# Analysis of Antioxidant Lipids in Five Species of Dietary Seaweeds by Liquid Chromatography/Mass Spectrometry

**DOI:** 10.3390/antiox11081538

**Published:** 2022-08-08

**Authors:** Siddabasave Gowda B. Gowda, Chen Yifan, Divyavani Gowda, Yui Tsuboi, Hitoshi Chiba, Shu-Ping Hui

**Affiliations:** 1 Faculty of Health Sciences, Hokkaido University, Kita 12, Nishi 5, Sapporo 0600812, Japan; 2 Graduate School of Global Food Resources, Hokkaido University, Kita 9, Nishi 9, Sapporo 0600809, Japan; 3 Graduate School of Health Sciences, Hokkaido University, Kita 12, Nishi 5, Sapporo 0600812, Japan; 4 Department of Nutrition, Sapporo University of Health Sciences, Nakanuma, Nishi-4-3-1-15, Higashi-ku, Sapporo 0070894, Japan

**Keywords:** seaweed, lipidomics, PUFAs, antioxidants, nutritional indices, liquid chromatography, mass spectrometry

## Abstract

Seaweeds are a good source of bioactive lipids and are known for their nutritional benefits, making them a valuable food source. Despite their dietary significance and nutritional importance, there are limited reports on comprehensive lipidome analysis of lipids with antioxidant properties. Therefore, this study aimed to compare the lipid profiles of five commonly consumed Japanese dietary seaweeds using non-targeted liquid chromatography/mass spectrometry (LC/MS). A total, of 304 molecular species from four major lipid classes were detected and characterized by MS/MS analysis. Multivariate statistical analysis revealed distinct lipid molecular compositions in kombu and sea mustard compared to hijiki, mozuku, and laver seaweeds. Kombu has been shown to contain large amounts of antioxidants, such as polyunsaturated fatty acids (PUFAs), and a high health promotion index compared to other seaweeds. Hierarchical cluster correlations indicated the predominance of glycerophospholipids (GPs) and glycerolipids (GLs) in sea mustard and kombu. As a result, dietary seaweeds have great potential as antioxidants and health-promoting foods for human consumption due to their high levels of PUFA-rich GPs and GLs. Unsaturated triacylglycerols are predominant in hijiki, whereas other health-beneficial lipids, such as monogalactosyldiacylglycerol and sulfoquinovosyl diacylglycerols, are predominant in sea mustard. This study provides a detailed characterization of lipids and their comparative fingerprints in seaweeds, demonstrating the potential use of dietary seaweeds in biotechnological and industrial applications involving the development of functional food products.

## 1. Introduction

Seaweeds have been used for centuries as a food source and in traditional medicines in East Asian countries [1,2]. In addition to their taste, they are also regarded as valuable sources of nutrition and health. The nutritional components of seaweed vary depending on various factors, including species, geographical location, temperature, season, salinity, light intensity, and interactions among these factors [3,4,5,6]. In general, they contain proteins, amino acids, carbohydrates, antioxidants, minerals, dietary fibers, vitamins, and lipids [7,8,9]. The use of seaweeds in the development of functional foods and nutrient extraction is widespread. Research on lifestyle-related diseases, such as cardiovascular disease (CVD) in the Japanese population, found that seaweed intake might reduce the risk of CVD and contribute to longevity [10]. A study by Murai et al. shows a comprehensive review based on the associations between seaweed intake and diabetes, weight reduction, blood pressure, cancer, and related factors in humans [10].

In recent years, foods enriched with seaweeds and seaweed extracts have drawn considerable interest because of their improved quality and several health benefits owing to their bioactive components, such as polyphenols, minerals, metals, and lipids. A high content of ω-3 polyunsaturated fatty acids (PUFAs) has been reported in seaweeds [11,12]. PUFAs play a crucial role in preventing type 2 diabetes, obesity, cardiovascular diseases, and cancer, and exhibit antimicrobial, antiviral, anti-inflammatory, and antitumoral properties [13,14,15,16]. PUFAs have been reported to be potential antioxidants. Hence, they are widely used in the development of food products with enhanced shelf life and quality [17,18]. Nevertheless, seaweeds also have complex lipids, such as glycerolipids (GLs), glycerophospholipids (GPs), and sterols [19,20], and display unique features, such as esterification with ω-3 fatty acids, including eicosapentaenoic acid (EPA) and docosahexaenoic acid (DHA) which are not found in terrestrial plants. GPs and GLs play structural roles in biological systems, including the formation of cytoplasmatic and chloroplast membranes [21]. Marine GPs have better bioavailability, resistance to oxidation, and a higher ω-3 PUFA content than lipids from other sources [22]. Moreover, they are better at delivering dietary ω-3 PUFA than terrestrial GPs, as already shown in several comparative studies [22,23,24].

Despite the great significance of seaweeds, they remain largely ignored, likely because of their low lipid content, high structural diversity, and complexity in analysis. Most previous studies on seaweed bioactive lipids focus on assays of total lipid extracts or enriched fractions. Only a limited number of studies focus on the identification and characterization of complex lipids at the molecular species level. Efforts have been made to profile lipids in seaweeds using techniques, such as gas chromatography/mass spectrometry [25], high-performance liquid chromatography [26], and nuclear magnetic resonance spectroscopy [27]. However, these techniques are time-consuming and provide limited knowledge about seaweed lipidomes. The lipidomics approach using the liquid chromatography/mass spectrometry (LC/MS) technique has received considerable attention because of its high sensitivity and comprehensive lipidomics. In this study, we aimed to obtain the lipid profiles of five commonly consumed Japanese dietary seaweed using a non-targeted lipidomics approach based on linear ion quadrupole-orbitrap mass spectrometry. Lipids with antioxidant properties and total lipid signatures were correlated and compared among these five species. Furthermore, the nutritional indices for each species were assessed based on their free-fatty acid levels and their lipid contents.

## 2. Materials and Methods

### 2.1. Materials

Five seaweed species, including brown (Hijiki (*Sargassum fusiforme*), Kombu (*Saccharina japonica*), Okinawa Mozuku (*Cladosiphon okamuranus*)), sea mustard (*Undaria pinnatifida*), wakame in Japanese), and a green Laver ((*Ulva lactuca*), nori in Japanese) types were purchased from different supermarkets in Sapporo, Hokkaido, Japan. All were commercially available and purchased in dried conditions, except for mozuku. Mozuku was dried at room temperature and crushed in a mixer. Crushed seaweeds were used directly for lipid extraction after weighing. The LC/MS grade methanol, isopropanol, and chloroform were purchased from Wako Pure Chemical Industries, Ltd., (Osaka, Japan). The mobile-phase additive ammonium acetate was purchased from Sigma-Aldrich (St. Louis, MO, USA). The EquiSPLASH Lipidomix quantitative standard for mass spectrometry and oleic acid-d9 were obtained from Avanti Polar Lipids (Ablabaster, AL, USA). The internal standard mixture was prepared using methanol by mixing the EquiSPLASH Lipidomix of 10 ng of each of the following: phosphatidylcholine (PC) (15:0-18:1(d7)), phosphatidylethanolamine (PE) (15:0-18:1(d7)), phosphatidyl-glycerol (PG) (15:0-18:1(d7)), phosphatidylserine (PS) (15:0-18:1(d7)), phosphatidylin-ositol (PI) (15:0-18:1(d7)), lysophosphatidylethanolamine (LPE) (18:1(d7)), lysophosphatidylcholine (LPC) (18:1(d7)), sphingomyelin (SM) (d18:1/18:0(d9)), ceramide (Cer) (d18:1/15:0 (d7)), triacylglycerol (TAG) (15:0-18:1(d7)-15:0), diacylglycerol (DAG) (15:0-18:1(d7)), cholesterol ester (18:1(d7)), and monoacylglycerol (MAG) (18:1(d7)) and 100 ng of oleic acid (d9).

### 2.2. Homogenization and Lipid Extraction

Five to six ceramic beads were added to a 2 mL Eppendorf tube preloaded with 100 mg dry seaweed (*n* = 3 each). The samples were homogenized using the Fisher Scientific Bead mill 4 (Fisherbrand, Tokyo, Japan) homogenizer for 2 × 30 s, followed by the addition of 1 mL of methanol, and subsequently, homogenization was repeated for one more cycle. The Bligh-dyer method was used for total lipid extraction in each seaweed sample using a single-phase, with minor modifications [28]. For every 100 µL of homogenized extract sample, 50% *v*/*v* of the internal standard mixture was added to a 1.5 mL Eppendorf tube and vortexed at 3500 rpm for 30 s. Subsequently, 100 µL of chloroform was added to the mixture, vortexed at 3500 rpm for 5 min and maintained at room temperature for 2 h. Finally, 20 µL of Milli-Q (ultrapure) water was added to the mixture, vortexed at 3500 rpm for 30 s, and centrifuged at 1500 rpm at 4 °C for 10 min. The supernatant was transferred to a new Eppendorf tube followed by vacuum drying.

### 2.3. Analysis by LC/MS

The vacuum-dried samples were redissolved in 100 µL of methanol, vortexed at 3500 rpm for the 30 s, and centrifuged at 4 °C at 1500 rpm for 10 min, and then transferred to LC/MS vials. The LC/MS conditions used in this experiment were similar to those used in our previous reports [29,30]. In summary, the Prominence UHPLC system (Shimadzu Corporation, Kyoto, Japan) was used for the chromatographic separation process with a standard automatic sampler and a ternary solvent delivery system. An Atlantis T3 C18 column (2.1 × 150 mm, 3 mm, Waters, Millford, CT, USA) was used for separation at a flow rate of 200 µL/ min. The mobile phases contained (A) Milli-Q (10 mM CH_3_COONH_4_), (B) isopropanol, and (C) methanol. A linear gradient was augmented as follows; negative mode: 0–1 min, 30% B, and 35% C; 1–14 min, 80% B and 10% C; 14–27 min, 85% B and 10% C; 27–28 min, 30% B and 35% C; positive mode: 0–1 min, 6% and 90% C; 1–10 min, 83% B and 15% C; 10–19 min, 83% B and 15% C; 19–19.5 min, 6% B and 90% C. For each run, the column temperature was maintained at 40 °C, and the injection volume was 10 µL.

Mass spectrometry (MS) analysis was performed using an LTQ Orbitrap mass spectrometer (Thermo-Fisher Scientific Inc., San Jose, CA, USA) in positive and negative ionization modes following previously established conditions [29,30]. The electron spray ionization source was maintained at a capillary temperature of 330 °C, nitrogen sheath gas flow of 50 units, and auxiliary gas flow of 20 and 30 units for the positive and negative modes, respectively. In negative ionization mode, the source and capillary voltages were 3 kV and 10 V, respectively, whereas, in positive ionization mode, they were 4 kV and 25 V, respectively. The MS^1^ scan range was set at *m*/*z* 160–1900 and 100–1750 for negative and positive ionization modes, respectively, in Fourier transform mode with a resolving power of 60,000 and collision energy of 35 V to obtain MS^1^ spectra for high-resolution masses. Low-resolution MS^2^ and MS^3^ spectra were obtained at a collision energy of 40 V in the ion-trap mode. The raw data were processed for alignment, peak extraction, identification, and peak area integration using MS-DIAL software version 4.2 (Yokohama, Japan). Identification of lipid molecular species was confirmed by high-resolution mass measurements and respective MS/MS spectra. Relative quantification of lipid molecular species was performed by considering the peak area ratios of the annotated lipids to the internal standard and multiplying it by the added internal standard. The concentrations were normalized to the weight of the seaweed used for extraction.

### 2.4. Statistical Analysis

The data were visualized in Microsoft Excel 2016, GraphPad Prism 8 software ( San Diego, CA, USA) as the mean ± standard deviation (analysis of each sample’s triplicate). One-way ANOVA with Tukey’s multiple comparisons test was employed (a value of *p* < 0.05) and was considered to be statistically significant. Principal component and cluster correlation analysis was performed using Metabo Analyst version 5.0 (https://www.metaboanalyst.ca/), accessed on 9 July 2022.

## 3. Results

**Figure 1:**
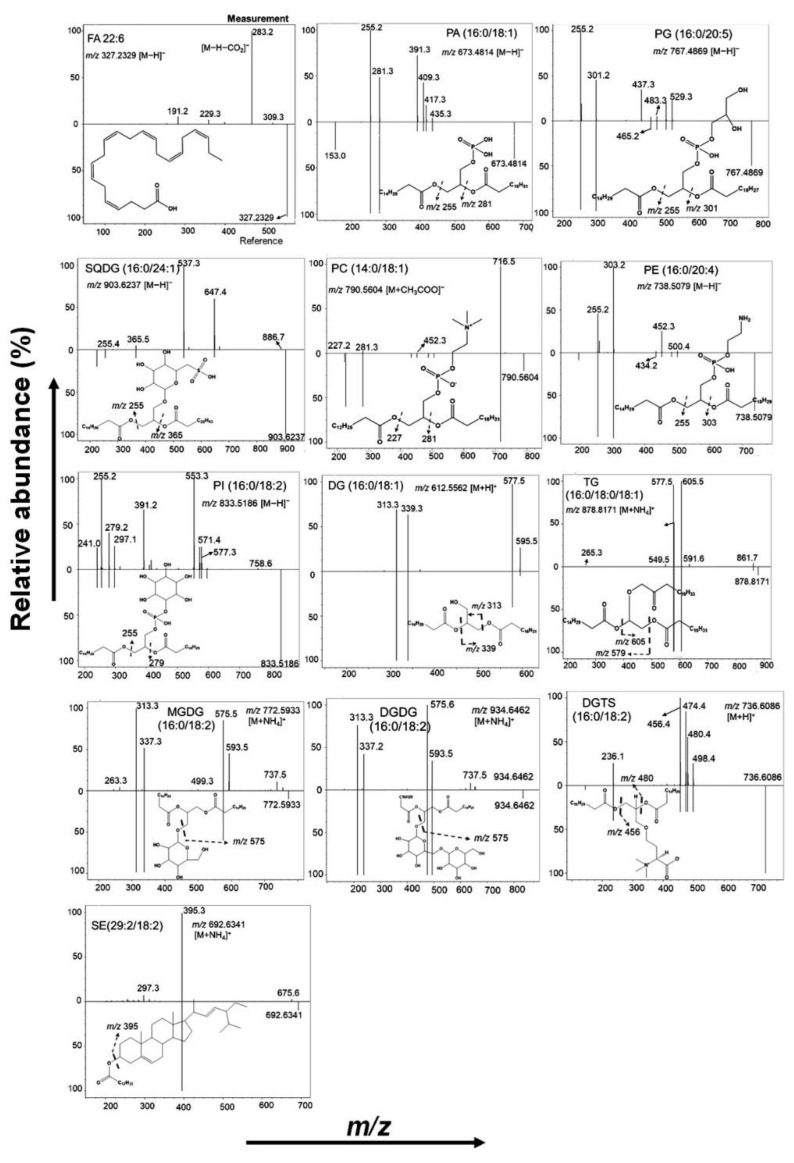
MS/MS spectra of representative lipid molecular species characterized in the study.

Untargeted lipidomic analysis of five species of dietary seaweeds has led to the detection of more than 300 lipid molecular species from four major lipid classes; fatty acyls (FAs), glycerophospholipids (GPs), glycerolipids (GLs), and sterols. Characterization of lipid molecular species was confirmed by high-resolution masses, retention time behavior, and MS/MS analysis using an inbuilt library of MS DIAL [31]. The MS/MS spectra of representative molecular species (experimental vs reference) are shown in Figure 1.

The measurement spectra were compared with the reference spectra to confirm the lipid structure. However, for some lipids, the reference spectra may not match exactly with the experimental spectra owing to the influence of various instrumental factors on the ion intensity. Therefore, it was not a necessary criterion for the ion intensity to exactly match the reference spectra. In the experimental spectra, a weak FA 22:6 [M-H]^−^ ion peak and a strong [M-H-CO_2_]^−^ ion peak is observed. Further loss of CO_2_ clearly suggests that the lipid was a polyunsaturated fatty acid. Lipids, such as FAs (FA 22:6), phosphatidic acid (PA (16:0/18:1)), phosphatidylglycerol (PG (16:0/20:5)), phosphatidylcholine (PC (14:0/18:1)), phosphatidylethanolamine (PE (16:0/20:4)), phosphatidylinositol (PI (16:0/18:2)), and sulfoquinovosyl diacylglycerol (SQDG (16:0/24:1) were detected and characterized in the negative mode as [M-H]^−^ or [M+CH_3_COO]^−^ ions. Diacylglycerols (DG (16:0/18:1)), triacylglycerols (TG (16:0/18:0/18:1)), monogalactosyldiacylglycerol (MGDG (16:0/18:2)), digalactosyldiacylglycerol (DGDG (16:0/18:2)), diacylglyceryl-trimethylhomoserine (DGTS (16:0/18:2)), and stigmasterol ester (SE (29:2/18:2)) were detected and characterized in the positive mode as [M+H]^+^ or [M+NH_4_]^+^ ions. The exact masses and plausible fragmentation patterns are indicated in each spectrum by the arrows.

**Figure 2:**
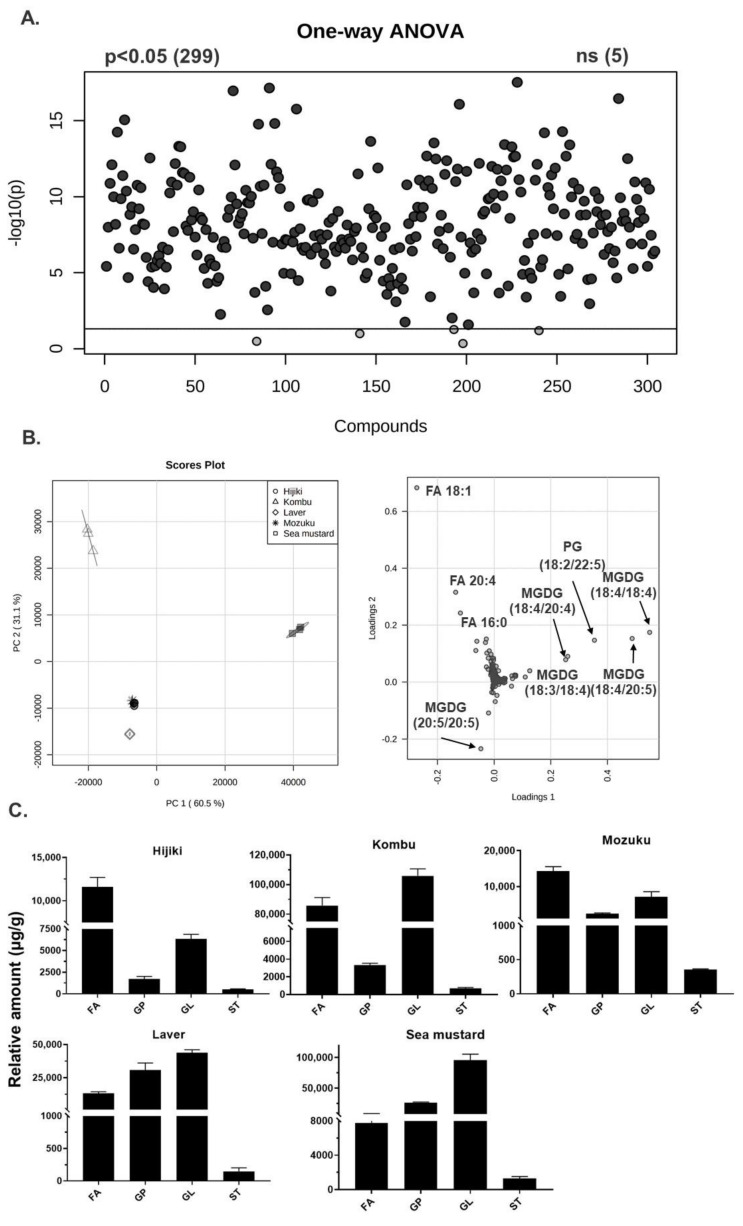
Multivariate analysis and distribution of lipid classes characterized in five species of seaweed. (**A**) One-way ANOVA analysis of all the lipid molecular species (Tukey’s HSD). (**B**) Principal component analysis score plots and loading scores of the lipids characterized in the study. (**C**). Comparison of the relative amount of major lipid classes determined in seaweeds. (Fatty acyls (FAs), Glycerophospholipids (GPs), Glycerolipids (GLs), and Sterols (STs)). ns: non-siginificant difference.

One-way ANOVA analysis and principal component analysis (PCA) results for all annotated lipid molecular species are summarized in Figure 2A,B. Most of the identified lipids are statistically significant (*p* < 0.05) as shown in Figure 2A. The PCA shows the differences between seaweed species based on the group lipidome changes. The loading score plot shows the distribution of the most significant variables (i.e., lipid molecular species) along the principal components and the groupings between the samples. PC1 and PC2 accounted for 91.6% of the total variance of the model and most of the variance was explained by PC1 (60.5%). The composition of lipids in all five species of seaweed shows group-specific clustering. While hijiki, mozuku, and sea mustard have almost identical lipid compositions, kombu and laver have different compositions among these three groups. According to the loading plot, this group separation was mainly attributed to the large positive or negative loadings of lipid molecular species, such as FA 18:1, FA 20:4, MGDGs.

**Figure 3:**
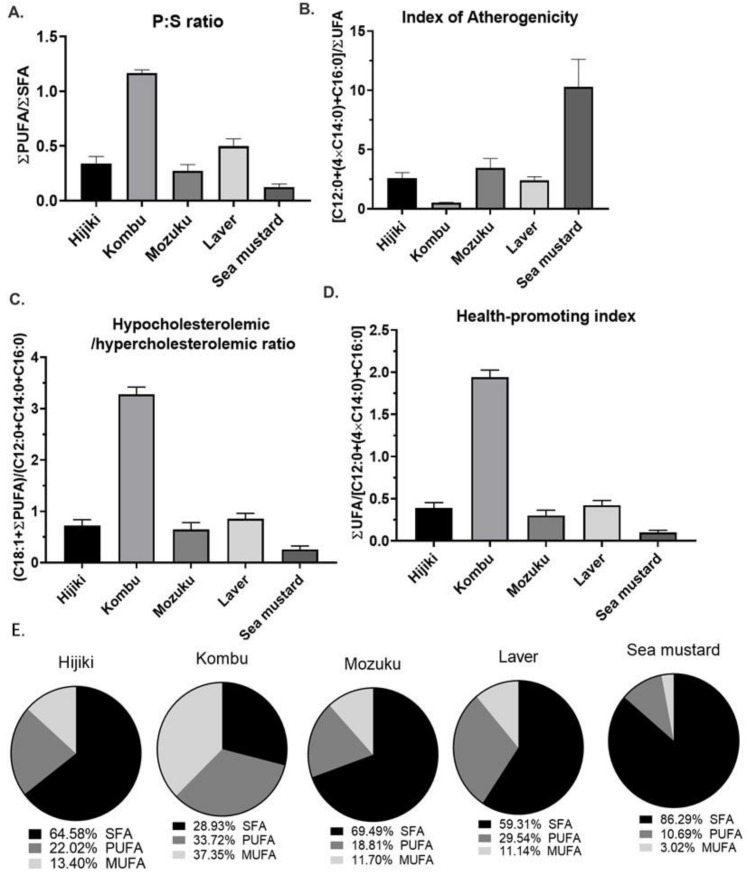
Nutritional indices and percentage distributions of free fatty acids. (**A**) Ratio of PUFA to SFA (P:S ratio). (**B**) Index of atherogenicity. (**C**) Hypocholesterolemic/hypercholesterolemic index. (**D**) Health promotion index. (**E**) Pie charts of percentage distributions of SFA, MUFA, and PUFAs among five types of seaweeds.

The relative amounts of each lipid class in all the five seaweed species are shown in Figure 2C. The number of FAs is higher in hijiki and mozuku, whereas GLs are predominant in kombu, laver, and sea mustard. Sterols are the least abundant lipids in all the species. Seaweeds are reported to show excellent nutritional properties owing to the abundance of GLs in seaweeds, such as kombu, sea mustard, and laver, including high levels of fiber in kombu, and our results are consistent with these reports [7,20,32,33,34]. Furthermore, the nutritional quality of the seaweeds was assessed based on the levels of free fatty acids using the formulas reported earlier [35,36]. The relative levels of fatty acids detected and characterized in seaweed are summarized in Table 1. Furthermore, Figure 3 shows a summary of indexes that can evaluate the nutritional quality of each type of seaweed. Figure 3A shows the polyunsaturated fatty acids to saturated fatty acids ratio (P:S ratio), which is an index normally used to evaluate the impact of diet on cardiovascular health [35]. The equation for this index is given below:

PUFA/SFA=∑PUFA/∑SFA.

It is designed under the assumption that all the PUFAs can suppress low-density lipoprotein cholesterol and contribute to lower levels of serum cholesterol, whereas all SFAs lead to high levels of serum cholesterol [35]. Therefore, the higher the ratio (1–1.5, a favorable range to reduce the cardiac risk), the more beneficial it is. The results show that kombu has the highest P:S ratio, whereas sea mustard has the lowest P:S ratio. This situation means that among the five species, kombu has the least risk of causing CVD, followed by sea mustard. However, Figure 3B shows the index of atherogenicity (IA), which reflects the atherogenic potential of FA. The formula used to calculate the IA is as follows:

$$\mathrm{IA}=\left[\mathrm{FA}12:0+\left(4\times \mathrm{FA}14:0\right)+\mathrm{FA}16:0\right]/\sum \mathrm{UFA}.$$

The index indicates the relationship between the sum of SFAs and the sum of unsaturated fatty acids (UFAs). In the equation, FA12:0, FA14:0, and FA16:0 are generally considered pro-atherogenic, whereas UFAs are considered anti-atherogenic [35]. Thus, the higher the index value, the higher the risk of atherogenicity. The results show that kombu had the lowest IA ratio, while sea mustard had the highest. This means that, among the five species, kombu has the lowest risk of atherogenicity, while sea mustard has the highest risk. Figure 3C shows the hypocholesterolemic/hypercholesterolemic index (HHI), which is used to calculate the effect of the FA composition on cholesterol. The formula is given below:

$$\mathrm{HH}=\left(\mathrm{FA}18:1+\sum \mathrm{PUFA}\right)/\left(\mathrm{FA}12:0+\mathrm{FA}14:0+\mathrm{FA}16:0\right).$$

The numerator (hypercholesterolemic) represents the FAs that lead to hypocholesterolemia, such as PUFAs and FA18:1 (cis or trans is omitted). The denominator represents FAs that lead to hypercholesterolemia, such as FA12:0, FA14:0, and FA16:0. This reflects the risk of CVD [35]. Therefore, the higher the index, the healthier the diet. The results show that kombu has the highest HH value, while sea mustard has the lowest. This means that among the five species, kombu is the least likely to cause high cholesterol, whereas sea mustard is the most likely.

Figure 3D shows the health-promoting index (HPI) designed to evaluate the nutritional value of dietary fat [35]. It is based on the effect of the FA composition on CVD. The equation is as follows:

$$\mathrm{HPI}=\sum \mathrm{UFA}/\left[\mathrm{FA}12:0+\left(4\times \mathrm{FA}14:0\right)+\mathrm{FA}16:0\right].$$

The formula shows that this is the inverse number of IA; therefore, the higher the index, the more beneficial the diet. It is generally used in dairy diets, however, in this study, we used it for seaweed. The results show that kombu has the highest HPI value, while sea mustard has the lowest. This means that among the five species, kombu is the least likely to cause high atherogenicity, while sea mustard is the most likely, as shown in Figure 3B. However, it should be noted that the HPI value was not the only deciding factor; other dietary factors also need to be considered when assessing the dietary importance of seaweeds. As shown in Figure 3E, kombu has the highest proportion of PUFAs (33.7%), while sea mustard has the lowest proportion (10.7%). PUFAs are essential for proper body development and normal metabolic functions. Long-chain PUFAs can act as antioxidants by scavenging free radicals and decreasing ROS production of reactive oxygen species in both in vitro and in vivo studies [17,37]. The health benefits effect of PUFAs (mainly ω-3) have been extensively reviewed in the recent literature [38], demonstrating the great potential of kombu as a source of PUFAs.

**Figure 4:**
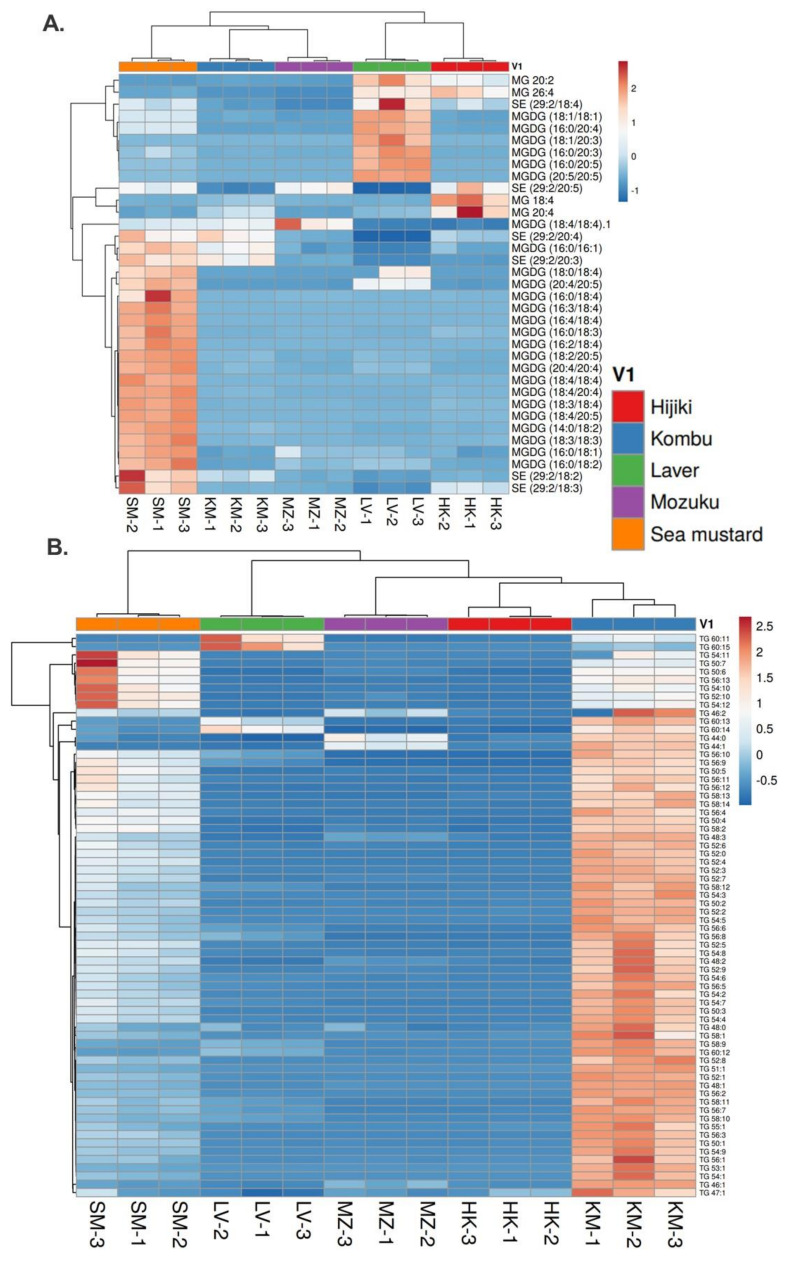
Hierarchical cluster correlation heatmap of glycerolipids and sterols. (**A**) Monoglycerols (MG), monogalactosyldiacylglycerol (MGDG), and stigmasterol (SE). (**B**) Triacylglycerols (TGs). HK: Hijiki, KM: Kombu, LV: Laver, MZ: Mozuku, SM: Sea mustard.

**Figure 5:**
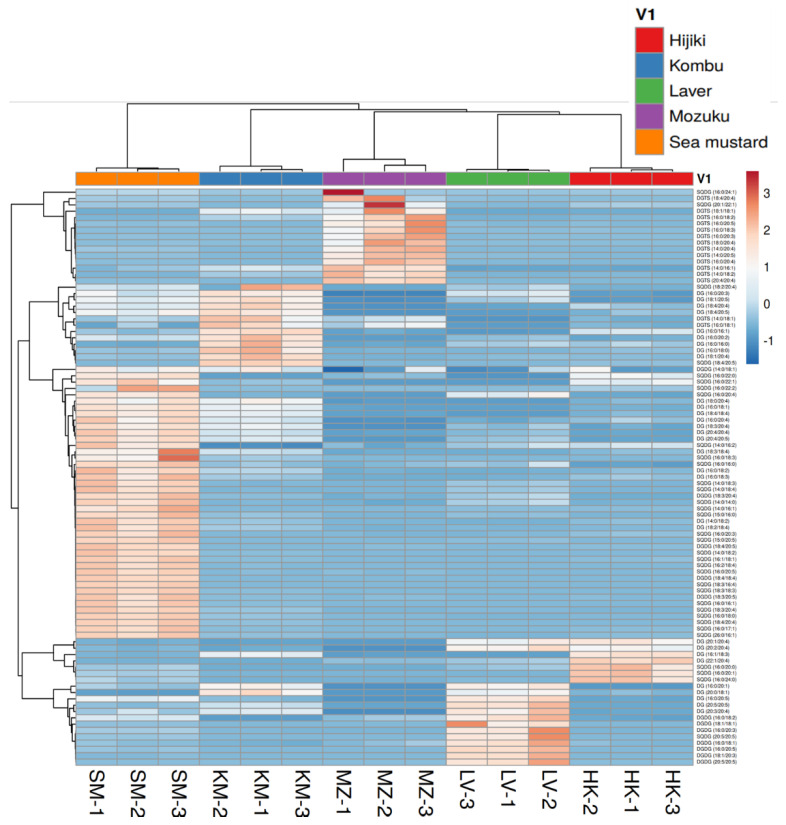
Hierarchical cluster correlation heatmap of glycerolipids including diacylglycerols (DG) and its derivatives, such as sulfoquinovosyl diacylglycerol (SQDG), digalactosyldiacylglycerol (DGDG), and diacylglyceryl-trimethylhomoserine (DGTS). HK: Hijiki, KM: Kombu, LV: Laver, MZ: Mozuku, SM: Sea mustard.

It is important to note that not all PUFAs are classified as essential fatty acids (EFAs), which cannot be synthesized in the human body, however, all EFAs belong to PUFAs. EFAs have two major groups: ω-3 fatty acids and ω-6 fatty acids. ω-3 fatty acids are well-known for their nutritional effects. Previous studies have shown that seaweeds have proved to be a valuable source of EFAs for human nutrition [39]. Furthermore, seaweed and its products have been considered to be important sources of PUFAs and play a significant role in food and nutritional applications [40]. The benefits of seaweed bioactive compounds, including PUFAs in various health and industrial sectors, including nutraceuticals, pharmaceuticals, biomedicine, cosmetics, agriculture and animal feed, were recently reviewed by Lomartire et al. [41]. Figure 4 and Figure 5, describe the hierarchical cluster correlation heatmaps of glycerolipids and sterols.

The MGDGs and SEs are relatively higher in sea mustard and laver (Figure 4A). TGs are abundant in kombu, followed by sea mustard (Figure 4B). They are the main components of very low-density lipoproteins and chylomicrons and act as transporters for energy and fat from food during metabolism [42]. Since the brain is unable to use fatty acids as energy sources, the glycerol in TGs is converted to glucose for use by the brain as an energy source [43]. Additionally, TGs are the main constituents of human body fat. Its energy density is twice as high as that of sugars and proteins, which means that kombu intakes may support a lifestyle with less glucose.

**Figure 6:**
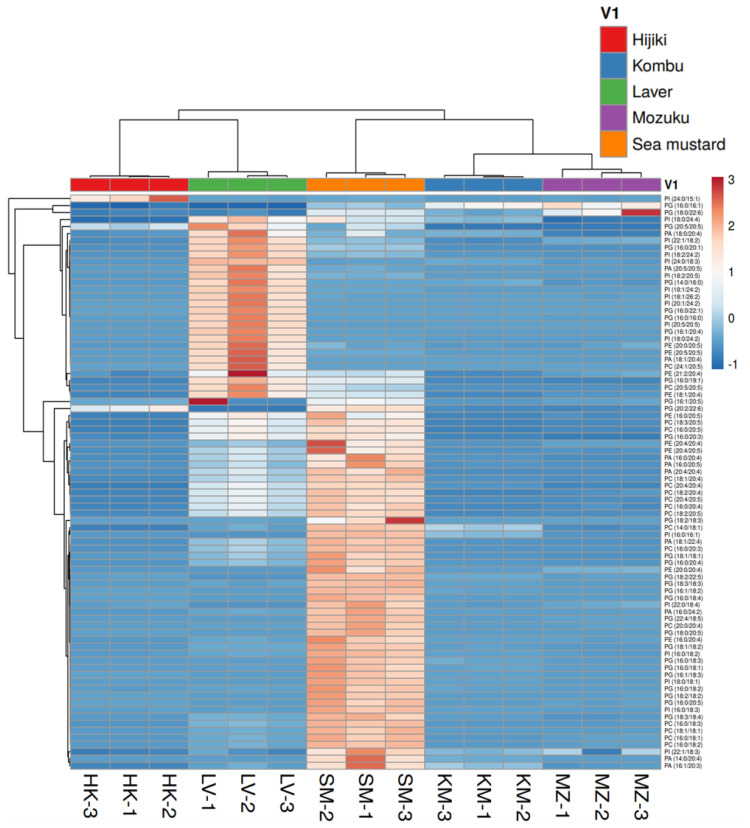
Hierarchical cluster correlation heatmap of glycerophospholipids. Phosphatidic acid (PA), phosphatidylglycerol (PG), phosphatidylcholine (PC), phosphatidylethanolamine (PE), and phosphatidylinositol (PI). HK: Hijiki, KM: Kombu, LV: Laver, MZ: Mozuku, SM: Sea mustard.

The results in Figure 5 show that sea mustard contains an abundant amount of lipids, such as SQDG and DGDG, whereas mozuku contains an abundant amount of DGTS lipids. SQDG, MGDG, and DGDG have been reported to have beneficial health properties and can inhibit DNA polymerases, which lead to tumor growth suppression [44,45]. MGDG is an active compound of the seaweed, *Sargassum cristaefolium* responsible for its anti-inflammatory and free-radical scavenging functions [46]. DGTS serves as a central intermediate in glycerolipid remodeling by maintaining EPA biosynthesis and providing the EPA acyl as a precursor for the formation of chloroplast glycolipids. Therefore, DGTS intake might be helpful for the biosynthesis of ω-3 FAs [47]. Our study limits the distinctions between ω-3 and ω-6 because the analysis was conducted in an untargeted mode and the quantitative results were not absolute. A hierarchical cluster correlation heatmap of the GPs is shown in Figure 6.

Complex lipids, such as PEs, PCs, PGs, PAs, and PIs are more abundant in laver and sea mustard than in hijiki, kombu, and mozuku seaweeds. These lipids play an important role in the synthesis of cell membranes. PEs plays an important role in membrane fusion and the breakdown of contractile rings during cell division [48]. PCs are the main components of biological membranes and act as pulmonary surfactants [49]. PIs play an important role in lipid signaling, cell signaling, and membrane trafficking [50]. PGs are glycerol-based phospholipids, that are the main components of biological membranes [51]. PAs are anionic phospholipids that are important for cell signaling and metabolic regulation [52]. Digestion and absorption of dietary GPs in the small intestine have been well studied [53]. Hence, the abundance of GPs in laver and sea mustard suggests that these two seaweeds have potential dietary benefits associated with the reported functions. Moreover, they are good sources of PUFAs. Despite the presence of various lipid molecular species, these lipids may easily deteriorate by hydrolysis and oxidation reactions, especially those which are abundant in PUFAs. Previous studies have demonstrated the effects of storage, temporal changes, and oxidative stability of lipids in seaweeds [54,55,56]. This study has some limitations because the amount of lipids obtained was relatively not absolute, and sample replicates were small. The indices were calculated based on free fatty acid levels and not on total fatty acids. This study lacks experimental data on the oxidative and hydrolytic stability of lipids in seaweed. Further, insights into the dietary effects of the presented seaweeds in animal models and clinical trials are necessary to understand their potential health benefits.

## 4. Conclusions

In conclusion, this is the first report to compare the lipid profiles of five different types of widely consumed Japanese dietary seaweeds using an untargeted LC/MS technique. Our analysis results suggested that seaweeds were highly enriched with antioxidant lipids, such as PUFAs, both in free form and with complex lipids, such as GPs and GLs. Interestingly, kombu was demonstrated to have a high health promotion index and an optimal P:S ratio, suggesting that it was a nutritionally important dietary seaweed. Sea mustard was characterized by a relatively higher amount of glycosylated glycerolipids than the other seaweeds. This study with lipid composition analysis and characterization of antioxidant lipids in seaweeds suggests their importance in the development of functional food products using industrial and biotechnological approaches.

## Figures and Tables

**Table 1:** Relative amount (µg/g) of free fatty acids including PUFAs detected in five species of seaweed. The data were shown as mean ± SD (*n* = 3). Two-way ANOVA with Tukey’s multiple comparisons test was applied and a value of *p* < 0.05 is considered to be statistically significant. The statistically significant FFAs are: FA 14:0 (ab,ac,bc,bd,be), FA 16:0 (ab,ac,ae,bc,bd,be,cd,ce,de), FA 18:0 (ab,ac,bc,bd,be,cd,ce), FA 16:1, FA 18:2, and FA 20:0 (ab,bc,bd,be), FA 20:1 (cd,de), FA 20:4 (ab,ad,ae,bc,bd,be), FA 20:5 (ab,ad,bc,bd,be,cd,de).

	Hijiki (a)	Kombu (b)	Mozuku (c)	Laver (d)	Sea Mustard (e)
Lipids					
FA 12:0	2.83 ± 0.06	4.8 ± 0.4	4.2 ± 0.2	2.8 ± 0.3	2.3 ± 0.3
FA 14:0	1943 ± 73	4425 ± 245	2757 ± 16	2517 ± 110	2217 ± 156
FA 15:0	44.3 ± 5.5	194.6 ± 9.3	51.4 ± 3.3	41.04 ± 4.64	7.79 ± 0.70
FA 16:0	2665.04 ± 277.13	13,674.72 ± 479.69	3435.35 ± 189.86	2622.20 ± 171.01	1562.28 ± 170.34
FA 16:1	234.86 ± 25.50	1260.67 ± 94.40	230.76 ± 35.13	63.21 ± 7.49	41.84 ± 9.46
FA 17:0	40.90 ± 0.80	3.75 ± 0.33	64.15 ± 3.19	44.94 ± 4.50	40.57 ± 4.02
FA 17:1	12 ± 3	120 ± 2	12 ± 2	5 ± 1	2.1 ± 0.5
FA 18:0	2605 ± 71	5143 ± 401	3350 ± 107	2533 ± 127	2439 ± 274
FA 18:1	807 ± 162	30448 ± 2796	1334 ± 344	507 ± 83	155 ± 80
FA 18:2	383 ± 80	6686 ± 378	711 ± 161	253 ± 42	107 ± 39
FA 18:3	23 ± 4	187 ± 2	8.6 ± 0.5	6.5 ± 0.2	7.25 ± 0.86
FA 18:4	27.7 ± 6.3	152.17 ± 2.40	5.45 ± 0.30	3.56 ± 0.15	24.78 ± 9.59
FA 19:0	8 ± 3	41.1 ± 6.8	13 ± 6	10.9 ± 1.1	13 ± 2
FA 19:1	5.2 ± 1.6	112.06 ± 8.19	10.3 ± 4.1	41.3 ± 8.4	1.2 ± 0.2
FA 20:0	174 ± 19	1321.16 ± 102.81	265.55 ± 57.03	60.58 ± 3.66	288.70 ± 29.27
FA 20:1	385.9 ± 102.6	79.27 ± 6.67	7.5 ± 3.9	673.66 ± 88.99	2.48 ± 0.18
FA 20:2	58.7 ± 14.5	339.1 ± 15.8	8.4 ± 1.5	319.32 ± 53.52	2.06 ± 0.60
FA 20:4	1524 ± 317	14972 ± 975	1175 ± 297	859 ± 160	372 ± 74
FA 20:5	531.13 ± 125.59	6575.12 ± 459.90	771.35 ± 159.21	2441.39 ± 386.84	311.02 ± 138.65
FA 22:0	0.27 ± 0.06	0.23 ± 0.05	12.23 ± 0.96	0.20 ± 0.04	0.30 ± 0.03
FA 22:1	0.15 ± 0.06	3.6 ± 0.2	11.76 ± 0.47	87.73 ± 11.74	0.81 ± 0.04
FA 22:4	2.92 ± 0.42	10.43 ± 0.39	4.24 ± 0.91	1.68 ± 0.44	1.31 ± 0.11
FA 22:5	0.9 ± 0.4	10.45 ± 0.89	0.36 ± 0.02	14.80 ± 3.44	0.51 ± 0.15
FA 22:6	0.49 ± 0.06	0.24 ± 0.05	9.32 ± 2.61	2.98 ± 0.50	5.13 ± 1.28
FA 24:0	0.1 ± 0.01	0.12 ± 0.03	0.73 ± 0.05	2.00 ± 0.64	141.61 ± 7.16
FA 24:1	103.75 ± 28.92	5.54 ± 0.32	1.22 ± 0.29	79.22 ± 8.19	5.15 ± 0.52
FA 26:0	0.63 ± 0.03	0.63 ± 0.21	0.17 ± 0.02	0.10 ± 0.03	0.18 ± 0.06
FA 26:1	4.51 ± 1.22	18.01 ± 1.47	68.39 ± 14.32	15.59 ± 2.95	26.04 ± 1.96

## Data Availability

Data is contained within the article.
